# Restricting the nonlinearity parameter in soil greenhouse gas flux calculation for more reliable flux estimates

**DOI:** 10.1371/journal.pone.0200876

**Published:** 2018-07-26

**Authors:** Roman Hüppi, Raphael Felber, Maike Krauss, Johan Six, Jens Leifeld, Roland Fuß

**Affiliations:** 1 Department of Environmental Science, Institute of Agricultural Sciences, ETH Zurich, Zurich, Switzerland; 2 Climate and Agriculture Group, Agroscope, Zurich, Switzerland; 3 Department of Soil Sciences, Research Institute of Organic Agriculture (FiBL), Frick, Switzerland; 4 Thünen Institute of Climate-Smart Agriculture, Braunschweig, Germany; USDA Agricultural Research Service, UNITED STATES

## Abstract

The static chamber approach is often used for greenhouse gas (GHG) flux measurements, whereby the flux is deduced from the increase of species concentration after closing the chamber. Since this increase changes diffusion gradients between chamber air and soil air, a nonlinear increase is expected. Lateral gas flow and leakages also contribute to non linearity. Several models have been suggested to account for this non linearity, the most recent being the Hutchinson–Mosier regression model (hmr). However, the practical application of these models is challenging because the researcher needs to decide for each flux whether a nonlinear fit is appropriate or exaggerates flux estimates due to measurement artifacts. In the latter case, a flux estimate from the linear model is a more robust solution and introduces less arbitrary uncertainty to the data. We present the new, dynamic and reproducible flux calculation scheme, kappa.max, for an improved trade-off between bias and uncertainty (i.e. accuracy and precision). We develop a tool to simulate, visualise and optimise the flux calculation scheme for any specific static N_2_O chamber measurement system. The decision procedure and visualisation tools are implemented in a package for the R software. Finally, we demonstrate with this approach the performance of the applied flux calculation scheme for a measured flux dataset to estimate the actual bias and uncertainty. The kappa.max method effectively improved the decision between linear and nonlinear flux estimates reducing the bias at a minimal cost of uncertainty.

## Introduction

For more than 30 years, trace gas emissions from soil have been measured with static (non-steady state) chambers, especially for greenhouse gases (GHG) like nitrous oxide (N_2_O). There are several guidelines available for the practical handling, chamber layout, experimental design and determination of concentrations within the chamber during deployment [1, 2]. To calculate a flux from the concentration measurements in the chamber headspace, researchers often simply use linear regression (LR) [3, 4]. Such LR-based flux estimates are least sensitive to measurement uncertainty caused by analytical limitations or variations due to gas sampling in the field [5]. However, the gas concentration in a static chamber theoretically follows a nonlinear shape during chamber closure because of underlying processes from diffusion theory and leakages [6]. When the nonlinear processes are ignored by applying a LR, a bias is introduced. This bias usually underestimates the flux depending on the extend of nonlinear effects in a current measurement setup. But on the other hand, estimating parameters for the nonlinear behaviour of a flux curve introduces uncertainty. Depending on chamber height, deployment time and soil physical properties (i.e. gas-filled porosity and bulk density), the estimated flux may increase with the assumption of a nonlinear behaviour relative to the more conservative LR [7]. Given a specific chamber system, uncertainty related to the flux calculation scheme is the largest single error source for the estimated flux [8]. The magnitude of this error has not been quantified reliably within the possible parameter space as it also depends on the chamber properties, measurement precision and the level of emissions.

Venterea [9] present a comprehensive summary of the available nonlinear flux calculation schemes (FCS) within the methodology guidelines by Klein et al. [1]. They list pro- and contra-arguments for the commonly used procedures (conventional FCS: Linear regression (LR), Hutchinson and Mosier (HM), Quadratic regression (QR); as well as advanced FCS: Non-steady state diffusive flux estimator (NDFE, [10]), hmr (R script by Pedersen et al. [11], based on the Hutchinson-Mosier equation) and chamber bias correction method (CBC by [12]). Their review shows that none of the methods can directly be applied to a measured flux dataset without additional tuning like manual screening with subjective decisions or arbitrary thresholds. Whereas LR can produce a considerable bias [11], all nonlinear estimates have large uncertainties for small fluxes [13] and deviation from the theoretical curvature. Venterea [9] modelled this deviation by switching on and off different processes in a soil diffusion model and compared the results to the available FCSs. They showed that under varying conditions most commonly used FCSs tend to substantially underestimate the theoretically modeled flux. Especially with increasing chamber enclosure time, nonlinear calculation methods perform better than linear estimates [14]. The different nonlinear approaches have considerable theoretical differences among themselves i.e. HMR has certain limitations with regard to its pseudo-steady state assumptions using Fick’s first law in a time-dependent model whereas the NDFE and CBC methods are based on non-steady state diffusion.

If one aims at reducing bias on flux estimates, the user has to combine a linear with a nonlinear method depending on the properties of each single flux measurement within the dataset. The hmr tool [11, 15] offers a manual screening of each flux and strongly recommends expert knowledge to choose between hmr, LR or zero flux estimates. Because this is subjective and not practical for large datasets, many users introduce some thresholds of certain indicators like *F*_*nonlinear*_/*F*_*linear*_ (g-factor) or statistical goodness of fit outputs like *R*^2^, *p*-values, standard errors (SE) or the Akaike information criterion (AIC, see [16]). The choice of these thresholds is arbitrary, barely justified and rarely documented in publications. Especially with the typical small sample size, statistical measures are biased or just not appropriate. A reliable criterion is needed that takes the flux strength into account and is robust to uncertainty of the concentration estimate by a specific measurement system. Most critical for nonlinear estimates are flux measurements that use only 4 time points with concentration measurements on a GHG gas chromatography system by offline manual vial sampling. This is however an often used sampling scheme. A commonly used calculation tool within the gas flux community of the German Soil Science Society (Deutsche Bodenkundliche Gesellschaft, DBG) is the R-package gasfluxes [17]. This tool offers different FCSs as well as an additional decision mechanism described in [18]. In addition to LR and hmr, a robust linear regression [19] is implemented to reduce the sensitivity of least square regression to outliers. Earlier selection schemes in the gasflux package chose between hmr, LR and robust linear depending on *p*-value and AIC of the fits and does not allow the absolute value of the nonlinear estimate to deviate by more than the g-factor (default is 4) from the absolute value of the linear estimate. Although this mixed FCS has been used in some studies [20–22] and is easy to implement, its performance for different systems has not been analysed systematically. Still many researchers are struggling to find a reliable method to use nonlinear GHG flux calculation schemes. Recent approaches came up with complicated decision trees using linear, quadratic and HM schemes at the same time depending on features in the chamber concentration values [23, 24].

There is no general rule or understanding for the thresholds and statistical decision criteria. It is very difficult for users of FCSs to estimate the impact of a certain FCS for their specific measurement system and how to choose the appropriate parameters. For this reason, we present i) a decision rule (kappa.max) that provides the best trade-off between uncertainty and bias a FCS introduces to the dataset based on ii) a tool that allows for a better understanding of the behaviour of the FCS used. We look for a relationship between measurement uncertainty (standard deviation of the GC system) and a dynamic threshold that allows to distinguish the LR and hmr regime in order to balance bias vs. uncertainty. The tool provides an automatic procedure for a safe use of nonlinear FCS also for inexperienced users. Consequently, commonly used decision trees could be pruned and simplified.

### Goals for optimised FCS decision rules

From visualising the behaviour of a certain FCS decision rule the appropriate procedure can be identified for a specific chamber measurement system. In general, an optimised scheme should fulfill the following goals:

1. The bias from the theoretically given nonlinear hmr shaped fluxes should be minimized but balanced to the estimated uncertainty. The importance of this goal depends on the purpose of the flux measurement, i.e. whether absolute fluxes need to be precise to come up with emission factors or treatments need to be compared among each other. For the latter the reduction of uncertainty (goal 4) may become more important than reducing bias (more details about goal 1 and 2 see [5]).
2. The uncertainty of the flux estimate should be minimized. We express the uncertainty in the simulated environment as difference between the 95% quantile and 5% quantile (IQ90). In the first step the uncertainty from the simulations is considered within the model framework and in a second step these uncertainties can be applied to the frequency of a real flux dataset on the model frame. In addition we estimated the mean squared error (MSE = bias^2^ + variance) in the model space to offer a balanced score between bias and uncertainty.
3. Arbitrary thresholds should be avoided because they are based on a subjective opinion of the user or the experience from a specific measurement system that may not be applicable to others.
4. An optimal decision scheme should be as simple as possible, i.e. use as few parameters as possible for decision making (pruning decision trees). A pragmatic and simple use means no additional measurements of temperature, bulk density or water content of the soil is needed; no expert knowledge about how to set the thresholds and which method to choose.
5. The calculation and the threshold parameters should be based on statistical principles or physical theories. If possible the parameter should have a meaningful unit and value.
6. The desired FCS should provide a smooth transition from small to medium to large *kappa* (the nonlinearity parameter, from being small means hmr can be used safely, to medium leading to a significant increase from linear to hmr estimate and to large describing a poorly defined nonlinearity where LR should be preferred). The threshold between the different regimes should be smooth as there is an uncertainty in any threshold value.
7. The detection limit (*f*_*det*_) should be small, i.e. similar to the estimate by linear calculation method.

## Methods

### Model framework for FCS visualisation and testing

In a first step, a simulation framework scans through a common range of curved chamber concentrations and flux strength. The hmr parameterization [11] of the Hutchinson-Mosier equation [25] for nonlinear flux estimates is used as approximation for any nonlinear flux curvatures.

$$\begin{array}{c} \text{C(t)}=\varphi +f_{0}\frac{exp(-\kappa \;t)}{-\kappa \;h} \end{array}$$ (1)

where

*C*(*t*) is the gas concentration at time *t*,

*φ* is the constant source concentration in a certain depth below the chamber (the chamber concentration converges towards this concentration, equal *C*(*t* = ∞),

*f*_0_ is the initial flux at time zero, when the chamber is closed hence the flux without chamber effect

*κ* (*kappa*) is the nonlinear shape parameter that is required to be > 0 and is estimated by the ordinary least squares method for −∞ < *f*_0_ < ∞.

*t* = time (after chamber closure)

*h* = chamber height

**Fig 1:**
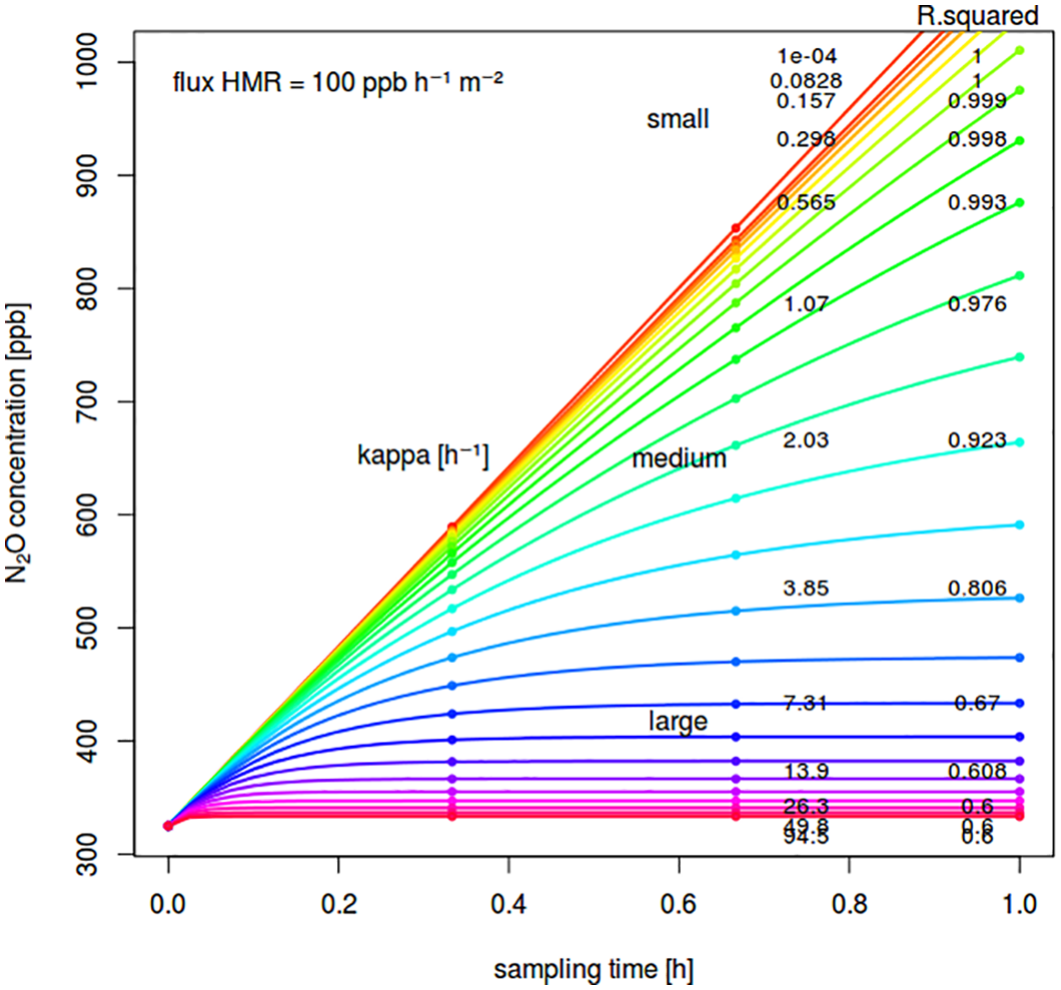
Example of simulated concentration-time curves depending on *kappa* and a specific flux size (chamber height: 0.14 *m*), *R*^2^ of a linear fit for the simulated concentrations are given for 4 sampling time points.

The hmr model was used to generate simulated data for a both logarithmic range of fluxes and nonlinear shape parameter *kappa* [11]. *Kappa* could be tentatively related to soil texture and moisture (dry/wet) that influence diffusion coefficients if assumptions are made regarding the depth of the gas source [9]. However, in this exercise we just scanned through a commonly observed range of *kappa* (Fig 1) without relating it to soil or chamber properties and the underlying diffusion models that themselves are also prone to bias and uncertainty [9, 11]. It was assumed that within the chosen range of *kappa*, the resulting concentration-time curves capture any diffusion characteristics of a typical soil-air system at different water levels. For the system specific input, a certain chamber area (i.e. 0.07 *m*^2^) and height (i.e. 0.14 m) were defined without loss of generality. A measurement device (i.e. gas chromatography GC) precision was then assumed as a constant standard deviation over the calibrated concentration range. Using this input data, a series (i.e. 25) of synthetic chamber concentrations were calculated for different flux sizes (i.e. *f*_0_; 0 − 5 *nmol* *s*^−1^ *m*^−2^) and nonlinear shapes (*kappa* from 10^−6^ to 10^−2^ *s*^−1^ logarithmic scale *n* = 25). Each of these replications followed the perfect hmr derived flux curve but in addition a random noise according to the GCs precision is added to each simulated gas sample concentration. The simulated sample concentrations were then fed back to the flux estimation script (i.e. gasfluxes by Fuß [17]) which uses the hmr equation (by Pedersen et al. [11]) again, applied with additional linear vs. nonlinear decision rules. In contrast to the original hmr implementation by Pedersen [11], gasfluxes uses a partial linear least square algorithm, which requires starting values for numeric optimization but is much faster.

For each grid point of the model space, a number of flux measurements (i.e. 50) with sample concentrations varying according to the GC precision (*sd*_*GC*_) were simulated for each set of parameters. A normal distribution with standard deviation of repeated measurements of an ambient standard was used to model GC precision. To our experience the precision of the GC is decribed best by a constant standard deviation, independent of the concentration level, especially for GC systems where ambient concentrations have a sufficient signal to noise ratio. To the resulting sets of simulated concentration-time points the fitting procedure was applied. The uncertainty of the flux estimated by any method can then be expressed as the IQ90 and plotted for the whole parameter space.

### Tested flux calculation schemes (FCS) on simulated data

Six different FCSs were selected that each apply different decision rules between linear and nonlinear regression for the interpretation of chamber flux data. The synthetically generated concentrations by the hmr equation (Eq 1) were then fed to the following calculation procedures (FCS). Note that Pedersens hmr approach [11] itself is a decision rule on its own, because it also uses linear regression for fluxes where no nonlinearity parameter (*kappa*) could be fitted. Especially with the added noise from measurement uncertainty, the hmr decision rule often cannot retrieve a flux even if concentrations were generated from the hmr equation. The methods presented here are either commonly used as calculation procedure in publications with static chamber flux datasets or they were suggested to offer robust solutions to decide between nonlinear and linear flux estimates:

- **LR:** This method applies linear regression (LR) by the least squares estimate.
- **RLM:** Robust linear regression method on the basis of the Huber M estimator is used as a less outlier-sensitive linear regression [19]. This function never weighs down the first or last time point with very few data points (min. 4).
- **HMR:** The nonlinear flux estimate by [11], *kappa* is estimated by minimising the mean squared error (MSE). If no local optimum is found for any *kappa*, the tool chooses a linear regression.
- **AIC:** The Akaike Information Criterion [16] can be used to compare the model quality and decide for the linear or hmr option that has the lower AIC score. Based on information theory AIC assesses the trade-off between goodness of fit and complexity of the model. It has been used for the selection between linear and hmr static chamber fluxes in the gasfluxes R-package [18, 26].
- **g-factor:** *F*_*nonlinear*_/*F*_*linear*_—the nonlinear flux calculation is not allowed to increase the linear flux estimate by more than a user-defined factor. The default for a maximum factor of 4 is used in the gasfluxes R-package and was used in [18]. This arbitrary chosen threshold concept can be transferred to a threshold of *R*^2^ or *kappa* [26] that allows for hmr estimates to be used. Many flux estimation procedures use this kind of fix and arbitrary threshold at a certain point.
- **KAPPA.MAX (the new FCS!):** see next section

### KAPPA.MAX—An improved flux selection method restricting HMR’s nonlinearity

Nonlinear hmr estimates are restricted by *kappa* being not allowed to exceed a certain threshold, depending on flux size (*f*_*lin*_), minimal detectable flux (*f*_*det*_) and measurement time (*t*_*meas*_):

$$\begin{array}{c} \kappa_{\text{max}}=\frac{f_{lin}}{f_{det}\;t_{meas}}, \end{array}$$ (2)

Eq (2) can be derived by expressing the hmr equilibrium concentration *φ* as *f*_*det*_ (see below). The kappa.max restriction follows the behaviour that *kappa* should be kept small when *f*_*lin*_ is small or *f*_*det*_ is large and vice-versa. Hence less impact of nonlinearity is allowed for small fluxes and for large uncertainties of the measurement system. The dimensionless ratio of these fluxes is divided by the measurement time that gives *kappa* the correct dimension (per time). The restriction of *kappa* depends on the ratio between minimal detectable flux and linear flux estimate.

**Mathematical derivation of KAPPA.MAX’s threshold value:**

$$\begin{array}{c} \varphi =C_{0}-\frac{f_{0}}{-\kappa \;h} \end{array}$$ (3)

Eq (3) defines the end concentration *φ* in the HM model (see [11]). *C*_0_ is the concentration at time zero.

$$\begin{array}{c} f_{det}=\frac{C_{f.det}^{t.meas}-C_{0}}{t_{meas}-t_{0}}\;h \end{array}$$ (4)

Eq (4) describes the (simulated) minimal detectable flux (*f*_*det*_) at specific chamber measurement time (*t*_*meas*_ = *max*(*t*)). There are different options to estimate *f*_*det*_ of a given system [1]. Our approach is described in the subsection “Estimation of *f*_*det*_”. The expression $C_{f.det}^{t.meas}$ is consequently the concentration at the latest measurement time point following emissions at the minimal detectable flux.

As a restriction of the nonlinearity parameter *kappa*, *φ* reflects the minimal detectable flux (*f*_*det*_) when *f*_*lin*_ = *f*_*det*_, hence when the linear flux is equal to the detection limit at *t* = *t*_*meas*_, *φ* is equal to $C_{f.det}^{t.meas}$. Eq (4) can be solved for Eq (3) (where *t*_0_ = 0):

$$\begin{array}{c} \varphi =C_{f.det}^{t.meas}=\frac{f_{det}}{h}\;t_{meas}+C_{0} \end{array}$$ (5)

Eq (5), *φ* expressed at the *f*_*det*_ boundary condition and insert the new expression for *φ* into Eq (3) where the linear flux estimate (*f*_*lin*_) serves as pragmatic flux estimate of the hmr equation (*f*_0_):

$$\begin{array}{c} \frac{f_{det}}{h}\;t_{meas}+C_{0}=C_{0}-\frac{f_{0}}{-\kappa \;h} \end{array}$$ (6)

That can be solved for kappa.max to get to Eq (2).

Limiting *kappa* as decision criteria has a behaviour similar to other parameters and could be translated into a certain *R*^2^ or a g-factor. By choosing higher and more pragmatic detection limits, one can decrease uncertainty for a minimal cost in bias.

#### Definition of KAPPA.MAX’s time factor *t*_*meas*_

The time factor *t*_*meas*_ relates the measurement time to *kappa*. *t*_*meas*_ should have the corresponding units as *f*_*det*_ and *kappa* (i.e. hour or second). We suggest to use the time difference between the first and last datapoint from the closed chamber. With this, all other parameters in kappa.max are derived during the flux calculation procedure.

#### Estimation of *f*_*det*_ (minimal detectable flux)

*f*_*det*_ was simulated with the input of the standard deviation of the GC measurements (*sd*_*GC*_ in *ppb*). For a pragmatic estimate of *f*_*det*_ the generated concentrations with the variability of the GC are calculated with the hmr scheme. A zero flux was prescribed on the hmr equation and concentrations following a random variation of *sd*_*GC*_ were generated (i.e. for 1000 fluxes). From the generated concentrations the hmr flux calculation was applied and the 97.5% quantile was used as *f*_*det*_ (similar to the approach of [13]. The higher the estimated *f*_*det*_ flux the less uncertainty by nonlinear flux estimates is introduced but at the cost of an increase in potential bias.

## Results

To validate the new approach with real measured data, we present a case study with data from [22]. The aim wis to choose the appropriate FCS decision rules depending on the properties of the measurement system (i.e. chamber size, sampling interval, measurement precision etc.).

### System specifications of a manual chamber measurement system

**Chamber size, measurement intervals, user input:**

Chamber volume (V): 0.014 *m*^3^

Chamber area (A): 0.07 *m*^2^

Sampling time (min): 0, 12, 24, 36 *min*

Precision of the measurement device (*sd*_*GC*_): 3 *ppb* SD (taking into account the handling of the vials, assumed to be constant for the usual concentration range, actual precision of the GC used by [22] rather between 1-3 *ppb*)

**Model input and assumptions** Atmospheric N_2_O concentration (c0): 325 *ppb*

Simulated flux (fmax): 0 − 5 nmol *s*^−1^ *m*^−2^ (logarithmic scale according the distribution of the measured fluxes)

Number of simulated flux levels: 25

Sequence of simulated *κ* (*kappa*): 10^−6^ to 10^−2^ *s*^−1^ logarithmic scale n = 25

Number of simulations per parameter combination (nMC): 50

Minimal detectable flux (simulated): 0.067 nmol *s*^−1^ *m*^−2^ (using 95% quantile for hmr calculated zero fluxes)

The chamber design of the sample dataset was described in [27] in detail. It consisted of a base ring that were permanently installed in the field and an opaque chamber with 30 cm diameter and 12 cm height. A stainless steel vent was installed for pressure equilibration. Additional rings could be installed between base ring and chamber to account for the actual plant height. In this case, a fan was installed to assure gas mixing in the larger chamber volume. Further details about the field experiment and sampling method can be found in [22]. The N_2_O flux measurements [22] provide an exemplary dataset from a reliable static chamber system. The almost 5500 fluxes were measured over more than two years from a very regular setup and under realistic agronomic field conditions. This dataset with its underlying chamber and GC setup were used to show the impact of the six different calculation methods described in the simulation framework.

**Fig 2:**
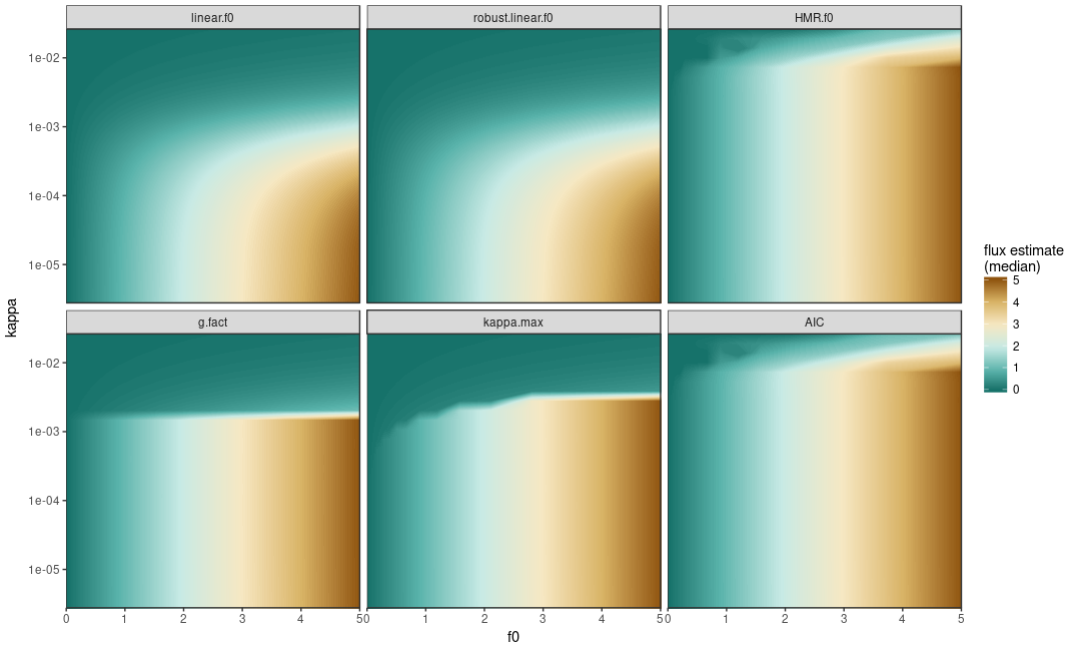
Median of the simulated fluxes for 6 different flux calculation schemes described in the method section. On the y-axis logarithmic values for *kappa* (about 10^−5.7^ to 10^−2^ *s*^−1^) are plotted against x-axis with predefined hmr flux (*f*_0_; 0-5 nmol N_2_O *s*^−1^ *m*^−2^). The colour code shows the median of the flux estimates for the given concentrations predefined by *f*_0_ and *kappa*. The assumed measurement uncertainty for simulations was 3 *ppb*, nMC = 50.

**Fig 3:**
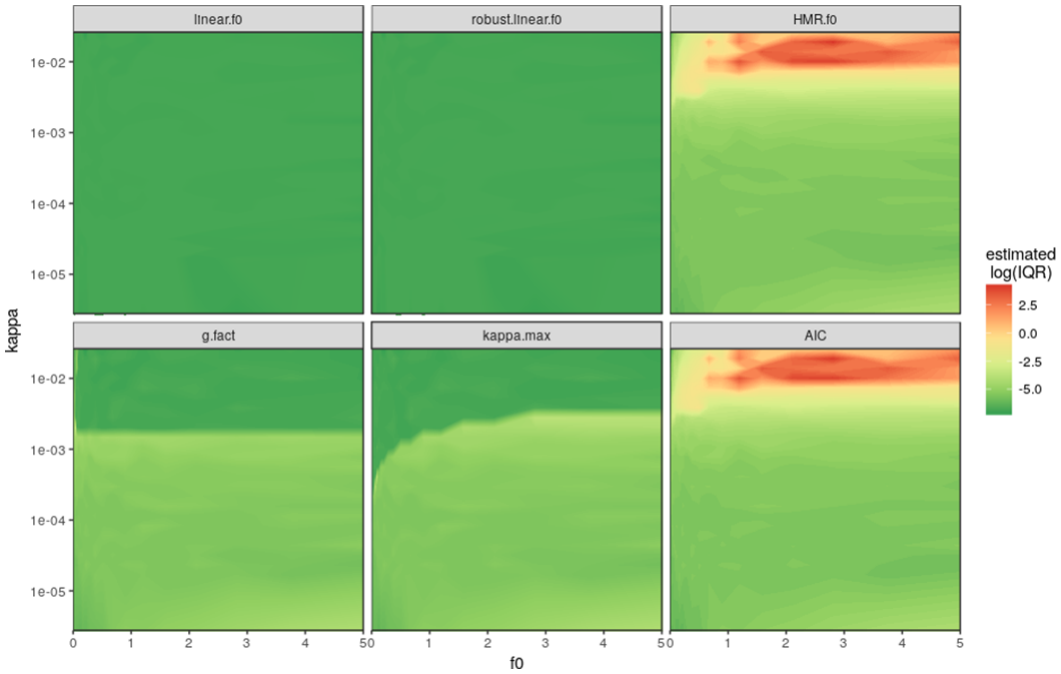
Uncertainty visualization as IQ90 of the simulated model space (same as Fig 2) for the 6 different methods shown on a logarithmic scaled colour code. Simulated measurement precision (*sd*_*GC*_) is 3 *ppb*, nMC = 50.

The medians (Fig 2) of the simulation results show that the linear model provide a smooth transition from large *kappa* (very high nonlinearity) to small *kappa* (close to linear behaviour). Whereas the uncertainties are stable (Fig 3) the bias increases with increasing *kappa*. This bias can be seen as drop in flux estimates from the predefined flux shown for small *kappa* values (at the bottom). In contrast the “always for hmr” decision reflects the prescribed nonlinear flux up to large *kappa* but gets unstable for *kappa* > 0.01 *s*^−1^. kappa.max accepts nonlinearity up to a certain point (roughly *kappa* = 0.001 *s*^−1^) and especially prefers linear estimates for small fluxes, depending on the measurement precision. The hmr and AIC methods look the same just as the ordinary linear and robust linear are roughly the same too (Figs 2 and 3). The g-factor decision criterion leads to a well defined break at a certain *kappa* value (in this case just above *kappa* > 0.001). It did not take into account that for small fluxes nonlinear regressions should be applied more restrictively.

There are large differences in the uncertainty for flux estimates between the different methods (note the logarithmic IQ90 scale). Especially for large values of *kappa*, the pure hmr estimate can get instable and introduce large variations to a dataset. Whereas linear estimates have a low IQ90 and hence uncertainty, hmr and AIC show much larger uncertainties especially with very high *kappa* values. kappa.max did balance the two approaches and reduced the uncertainty (Fig 3) and bias (Fig 2) considerably. Any introduction of nonlinear hmr estimates increases the uncertainty tremendously. However, the scope of this uncertainty is driven by the precision of the measurement instrument (see *sd*_*GC*_).

### Model application to the measured flux dataset

After exploring the model space in the relevant range of *kappa* and flux sizes, we apply the different calculation methods to the introduced dataset of a two years field measurement campaign [22]. The results of the different calculation methods are shown in Table 1. For the total number of 5470 fluxes the Table shows for each method its resulting geometric mean flux, the number of fluxes that used hmr, the relative difference to the linear calculation (mean flux method / mean flux linear), the mean IQ90, bias (deviation from HMR estimate) and MSE (squared bias + variance) estimate. The last column in Table 1 shows the estimated *f*_*det*_ from the 0.975 quantile (95% CI) of 1000 simulated zero fluxes and a *sd*_*GC*_ of 3 *ppb* for each method.

**Table 1:** Effect of different method of gas flux calculation on the example dataset (total number of fluxes is 5470), with estimated mean IQ90, bias and MSE having same unit as the mean flux and *f*_*det*_.

FCS	Mean flux	# hmr fluxes	deviation from	mean IQ90	mean bias	mean MSE	*f*_*det*_
Method	[*nmol* *s*^−1^ *m*^−2^]	selected	linear estimate	estimate	estimate	estimate	[*nmol* *s*^−1^ *m*^−2^]
LR	0.197	0	1.00	1.15E-3	-8.71E-2	5.99E-3	0.028
RLM	0.197	0	1.00	1.16E-3	-8.71E-2	5.98E-3	0.028
hmr.f0	0.719	3271	3.64	7.08E-3	-5.89E-2	4.68E-2	0.052
AIC	0.714	2656	3.62	7.06E-3	-5.90E-2	4.68E-2	0.033
g-factor	0.241	3001	1.22	4.90E-3	-6.05E-2	2.93E-4	0.040
kappa.max	0.221	1925	1.12	3.28E-3	-6.62E-2	3.76E-4	0.028
RF2017 [26]	0.323	3248	1.64	6.07E-3	-5.89E-2	6.77E-5	0.046

When the methods simulated in Figs 2 and 3 are applied on a representative large flux dataset [22], the geometric mean flux varies from +0% for robust linear to +264% if hmr and/or AIC is used (Table 1). The difference in the mean flux is related to the number of hmr fluxes selected, but between methods there are considerable differences in which fluxes are effectively selected. The large deviation from the linear flux in the hmr and AIC shows the urgent need to restrict the use of hmr. But apparently the AIC is not an appropriate criterion because the choice is too relaxed towards hmr. With an increase of only 12% from linear methods kappa.max has the smallest deviation compared to all other nonlinear schemes. The few percent difference in the overall mean of the dataset is still considerable because this is the effect of a few major fluxes that dominate the dataset. The additional method ‘RF2017’ (not shown in Figs 2 and 3) is an example for an actual use of the gasfluxes package published recently. ‘RF2017’ chooses nonlinear regression if *kappa* < 20 as arbitrary chosen threshold [26]. The method shown in [22] indicated a 20% increase from the linear to nonlinear estimate of N_2_O but this is derived from a two months shorter dataset and results from interpolated cumulative emissions. The IQ90, bias and MSE estimates are calculated from the measured flux dataset projected on the simulation grid to its corresponding *kappa* and flux level, assuming the original fluxes are actually based on the model input parameters. The IQ90 is the smallest for the linear methods, while roughly two times the linear IQ90 for kappa.max and hmr/AIC have almost 6 times higher uncertainties in this dataset than linear estimates. Looking at the estimated bias, ‘RF2017’ achieves the lowest bias whereas the linear methods have the largest negative bias. In combination of bias and uncertainty, the MSE shows that kappa.max scores the middle value together with the g-factor method. Finally, the minimal detectable flux of kappa.max is as small as the linear methods, whereas all other ones result in much higher uncertainty for very small fluxes.

**Fig 4:**
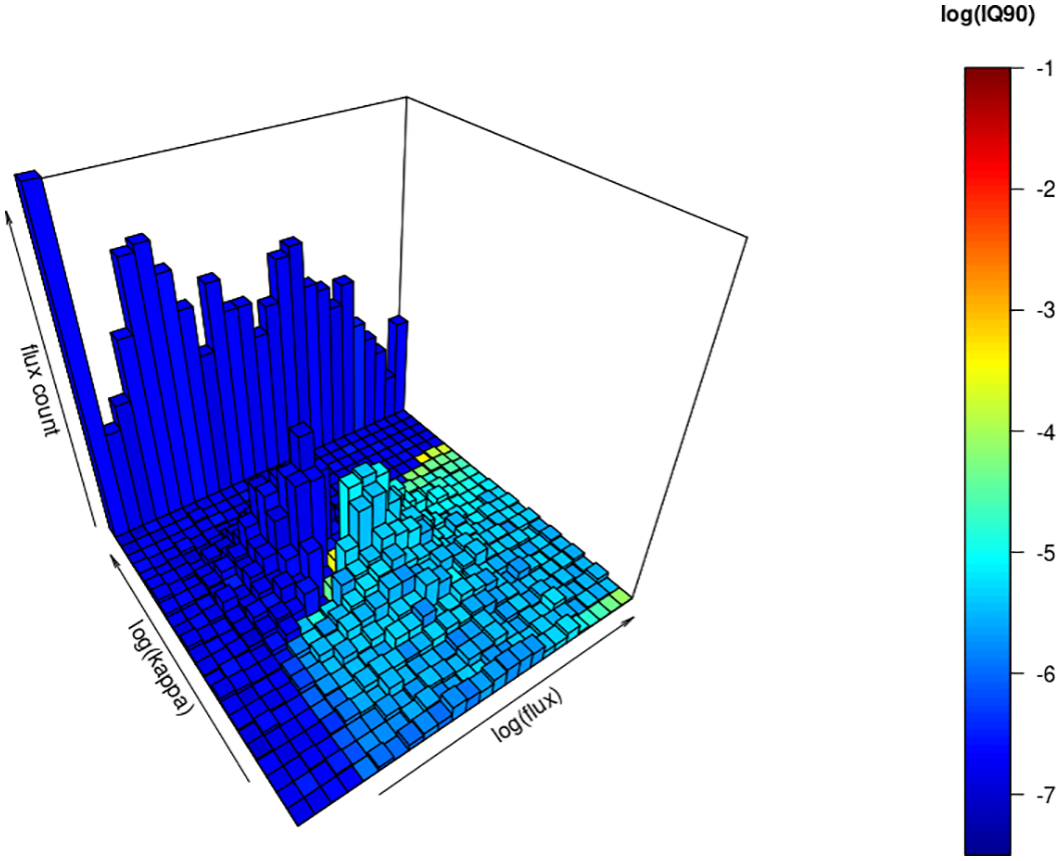
Histogram of the flux dataset projected onto the modeled *kappa*/*f*_0_ space with shading of the log(IQ90) using the kappa.max method. Fluxes larger than the simulation range (> 5 *nmol* *h*^−1^ *m*^−2^) are summed up in the largest flux column on the upper-right side. Where hmr could not be fitted, the fluxes are counted for the largest *kappa* value (upper-left). Whereas the range and units on the kappa and flux axis are the same than for antecedent figures, the z-axis with the flux counts within the range of the model grid reaches from 0 to 160 counts.

Fig 4 visualises the fluxes from the dataset as histogram in the model space (already shown in Figs 2 and 3). Bar height indicates the number of fluxes that were measured within the range of flux sizes and *kappa* determined by the model space. Fluxes that exceeded the modelled range (> 5 nmol *h*^−1^ *m*^−2^) where set to the maximum value available on the simulated framework (and show up in upper right row). This gives a sense of what the impact of the large fluxes to bias and uncertainty potentially is. If *kappa* could not be fitted the fluxes are counted in the row of the largest modelled *kappa* (showing up with low uncertainty at the upper-left row). The uncertainty associated with these fluxes is small, because they are calculated with linear regression. The colour code shows the uncertainty as logarithmic IQ90 of the simulation for the kappa.max method (see Fig 3). The kappa.max decision algorithm separates the fluxes that could be fitted by hmr and the ones that could not. This reduces the uncertainty of the dataset considerably. On the left (dark blue colours) are the nonlinear fluxes that are not trusted whereas on the right (light blue to yellow) they are trusted with a mostly acceptable modelled uncertainty. With this visualisation, critical fluxes (yellow-red colours) can be identified that need special attention.

### KAPPA.MAX’s sensitivity to precision and measurement time

**Fig 5:**
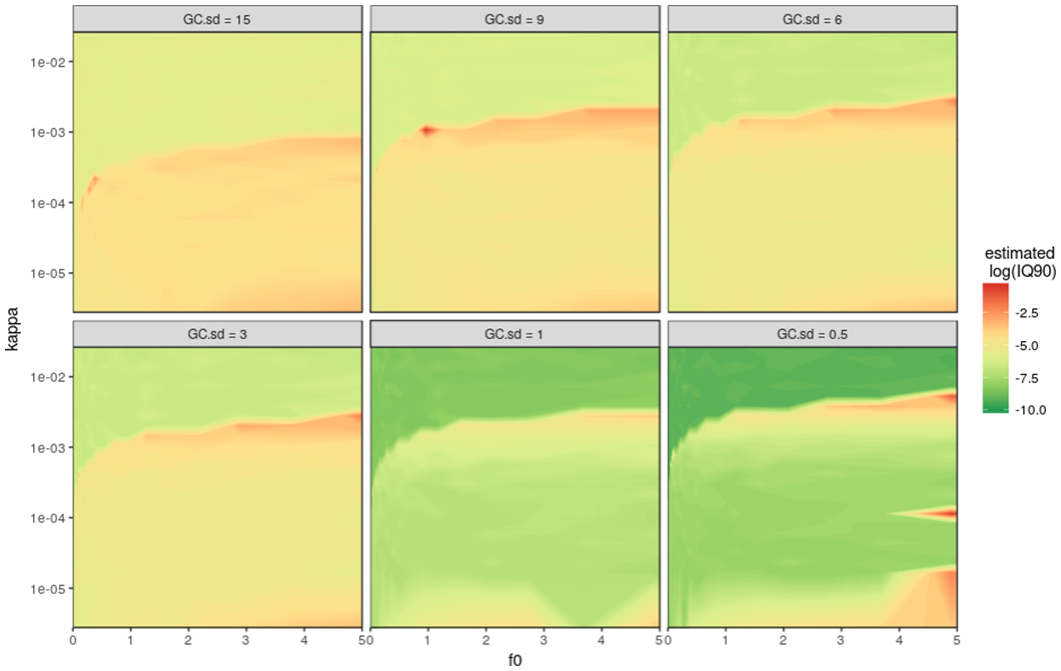
Sensitivity analysis of measurement precision (*sd*_*GC*_ = standard deviation of a GC measurement) using the kappa.max calculation method in the simulation framework. The six plots use *sd*_*GC*_ values of 0.5, 1, 3, 6, 9 and 15 *ppb* to calculate the minimal detectable flux (*f*_*det*_) that is then used to restrict *kappa* estimates of hmr. t(max) = 60 *min* for all plots.

**Fig 6:**
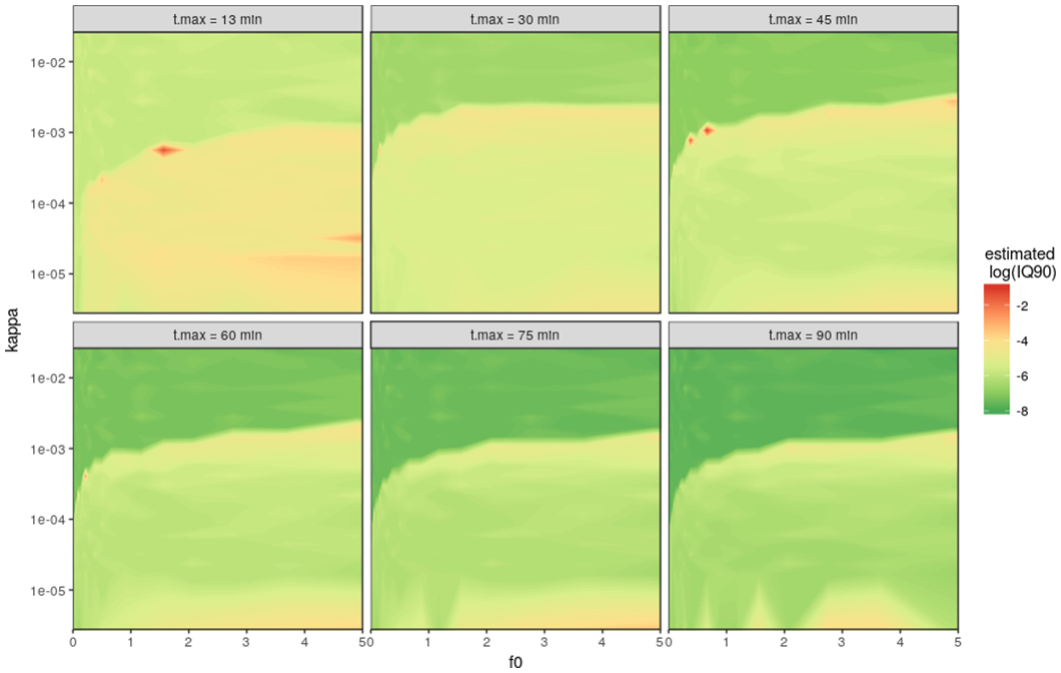
Sensitivity analysis of the maximal chamber time (t.max = time of the final gas sample—t(max)) using the kappa.max calculation method. The six plots use t(max) values of 13, 30, 45, 60, 75 and 90 minutes to calculate maximal allowed *kappa* in the hmr flux estimate. *sd*_*GC*_ = 3 *ppb* for all plots.

The driving parameters for the kappa.max hmr-restriction function are the measurement precision *sd*_*GC*_ and the chamber measurement time *t*_*meas*_. Figs 5 and 6 show a series of different values for these parameters tested in the simulated model framework introduced above. The *sd*_*GC*_ was used to simulate the minimal detectable flux (*f*_*det*_, see method section) that is then used in the quotient with the linear flux estimate to restrict hmrs nonlinearity parameter *kappa*. Also *sd*_*GC*_ is used for all simulated concentrations of the model framework. Fig 5 shows that *sd*_*GC*_ influences both the uncertainty for the linear as well as nonlinear estimates. It also impacts the threshold where the algorithm switches from hmr to linear estimates. For smaller measurement uncertainties hmr fluxes are trusted up to higher *kappa*, whereas for higher *sd*_*GC*_ linear estimates are favoured.

Similar to *sd*_*GC*_, *t*_*meas*_ as the duration of the chamber measurement changes the uncertainties for the linear and nonlinear flux estimates, as well as the threshold line between the two (Fig 6). Because the same measurement uncertainty is used, this variation is distributed among a shorter time of measurement, hence the shorter the chamber time, the higher the uncertainty of the flux estimate. This applies to the linear regression as well because the elapsed time increases the robustness of the fit.

## Discussion

The newly introduced approach to improve balanced estimates for static chamber fluxes, kappa.max, offers substantial improvements compared to other methods used in literature [1]. Most importantly our data shows, that large deviations (> 20% increase) from the linear estimates are questionable. Furthermore the kappa.max method avoids the need for expert knowledge and arbitrary thresholds. Introducing the minimal detectable flux to the decision strengthens the practice to actually calculate and report the precision of a measurement system. The potential bias and uncertainty introduced by the flux calculation can be estimated with the presented simulation framework. Its calculation will be implemented into the R-script. Bias and uncertainty can thus be reported in upcoming studies accordingly. The different methods for the flux calculation highlight the driving factors of a static chamber system. The gas sampling scheme needs to be optimised for the expected flux size depending on chamber dimensions. The model framework can be used to simulate and visualise the impact for the specific parameters of a certain measurement system. After visualising the performance of the flux calculation method for the system (Figs 2 and 3), the user can also check in which region of the *f*_0_/*kappa* space the data is gathered (Fig 4).

### 1. Bias

Linear or robust linear schemes have a large bias that is stable over the tested range of precision. In our simulation framework a zero bias cannot be achieved because the hmr algorithm itself collapses with increasing *kappa* estimates (sharp increase in uncertainty, see Fig 3). According to how the FCS bias was estimated, the decision rules perform in the order: hmr > AIC > g-factor > kappa.max > robust linear = linear (Fig 2 and Table 1). kappa.max is the only procedure that varies its bias with measurement precision. Clearly, one cannot simply aim for the lowest bias in this framework, because large *kappa* cannot be estimated reliably and the flux can be overestimated excessively.

### 2. Uncertainty

The smallest uncertainty is achieved by the linear or robust linear flux estimate. The use of hmr needs restrictions to prevent unstable estimates which introduce a large uncertainty to the dataset. The method ranking with respect to uncertainty is: linear = robust linear > kappa.max > g-factor > AIC = hmr. Our results imply that the AIC is a too relaxed criteria for the linear-nonlinear decision and does not sufficiently reduce the uncertainty from the hmr approach. The g-factor (<= 4) method seems to be a reasonable threshold for this system, but does not change with measurement precision. Also it does not take into account that smaller fluxes at critical levels of *kappa* need to be treated with more caution than larger fluxes. The reduction in uncertainty is provided at a comparable low cost of bias with the proposed kappa.max scheme.

### 3. Arbitrary thresholds

kappa.max was developed to overcome the frequently used approach to apply arbitrary thresholds based on expert knowledge. However, many studies used thresholds for *R*^2^ (i.e. > 0.8 in [24]), *kappa* (i.e. *kappa* < 20 *h*^−1^ in [26] or g-factor < 4 [18, 22]) that are arbitrarily chosen from some experience. Our simulation showed that these thresholds have a similar effect on the nonlinear flux selection and can consequently be translated into each other (i.e. for the system at a *sd*_*GC*_ of 3 *ppb*, the results are similar for *R*^2^ < 0.8; *kappa* < 5 *h*^−1^ and g-factor < 4.5). Also statistical performance indicators like AIC, *p*-value or SE are rather arbitrary because it is not clear how these statistical expressions should decide between linear and nonlinear calculation. The newly suggested kappa.max method tries to replace the arbitrary threshold by applying a dynamic one that is derived from important parameters of the measurement system. The kappa.max approach shows that such a more reasonable threshold leads to better results than the arbitrary ones and is furthermore based on physical logic.

### 4. Simplicity of the method

In terms of simplicity the ranking of the screened methods is: LR > robust linear > hmr > AIC = g-factor > kappa.max (> CBC, [12]). With less statistical assumptions the linear approach is the most robust. The less additional information is needed the easier it can be applied. However when nonlinear effects dominate the concentration measurement in a chamber, the measurement precision should be used as additional input to balance bias and uncertainty for nonlinear regressions (like kappa.max). A system with an unknown measurement precision cannot handle the optimal choice between linear and nonlinear schemes. In contrast the CBC (see introduction, [12]) would additionally need measurements of the soil water content and bulk density from the field, thereby introducing additional sources of uncertainty and bias. Another recent example of a highly complex flux calculation scheme was suggested by [23]. Similar to [24] they developped a flow chart that distinguishs between different flux calculation methods depending on certain patterns in the chamber concentration measurements. In contrast our results show that a dynamic threshold for *kappa* allows for a nonlinear approach for the whole range of fluxes, especially around the minimal detectable flux.

### 5. Calculation based on statistical principles and physical theories

Although hmr equations reflect the diffusion from a constant source concentration *φ* in depth *d* below soil surface, they don not represent a sophisticated diffusion model. Venterea [9] describes the deviation of nonlinear FCSs towards the theoretical curvature. Compared to the other methods tested, hmr has an average performance and generally underestimates the theoretically modeled flux. The advantage of hmr is that it can represent very small to larger deviations from the linear estimate with the estimation of just one parameter (*kappa*). Fitting *kappa* to the concentration measurements estimates any combination of physical effects and soil heterogeneity, that lead to a flattening of the concentration increase. In addition to the decreasing concentration gradient, chamber leakage or lateral gas transport in soil leads to a reduced slope with time. These effects are influenced by the diffusivity of the soil that is again driven by the water content. That is why approaches like the CBC [12] use data about soil water content and soil bulk density. However, we expect that the hmr deviation from theoretical diffusion models only introduce a minor bias to the flux estimates. Rather factors like the measurement precision, vial handling and random fluctuation in the chamber concentration from wind gusts etc. superimpose model deficiencies [3, 28]. The theoretical diffusion models themselves are just approximations and cannot describe the dynamics below the chambers in detail (i.e. cracks, bioturbation or soil heterogeneity in general). Using the hmr procedure allows the numerics to fit the *kappa* parameter to match all these unknown factors best to the measurements. The challenge is to find the limits to the underlying assumptions and consequently use LR for those cases, as it is suggested by the kappa.max method. In comparison to g-factor and AIC, kappa.max has a stronger link to physical parameters because the precision can be measured objectively.

### 6. Smooth transition between nonlinear and linear regression methods

Linear methods perform well with respect to a smooth transition from nonlinear to linear estimates. Most other schemes suddenly drop from hmr to linear and create large uncertainty. By using our simulation framework and a histogram of the flux dataset (Fig 4) it should be verified if a significant number of fluxes lies close to a sudden transition between linear and nonlinear regimes. The uncertainty in the estimate of hmr’s *kappa* can introduces variations of several 100 percents of the flux estimate. kappa.max leads to a better defined separation of linear and nonlinear fluxes (Fig 4).

### 7. Low detection limit

The detection limit is related to the uncertainty for low fluxes. The most stable approach is using linear or even a robust linear regression. A combination of linear and nonlinear schemes needs to detect fluxes close to detection limit and appoint them to linear regression. The compared methods perform in terms of low detection limit as follows: robust linear = linear = kappa.max > AIC > g-factor > hmr (Table 1). Note that the original *f*_*det*_ used to calculate kappa.max’s dynamic threshold, is derived from the hmr method. kappa.max methods perform as good as the linear ones, whereas AIC seems to be superior to g-factor or hmr. [13] has studied the effect of the flux calculation regression on detection limits in detail and found that quadratic regressions had a lower detection limit than hmr as well. This is mainly because the hmr method urgently needs additional restrictions especially for small fluxes.

The gasfluxes package offers a simulation function for the detection limit that is similar to the approach of [13].

### Limitations and further needs for research

The hmr equations are able to fit a nonlinear flux estimate assuming certain diffusion conditions. But there can be a considerable theoretical flux underestimation by the Hutchinson-Mosier model as shown by [12] and [9]. Models that are more closely linked to detailed soil gas diffusion processes suffer in practice from unmet assumptions like one-dimensional vertical diffusion in the soil profile or that soil is vertically uniform with respect to physical properties [9]. However, one could try to involve measurements of soil water content and use this information to improve the kappa.max decision accordingly. It needs to be explored systematically how soil water content influences the nonlinearity parameter *kappa*.

We showed that a combination of linear and nonlinear flux calculation by the use of the kappa.max restriction equation (Eq 2) is a very practical and pragmatic approach. There is still a need for further testing with different system, especially for datasets with more than 4 data points in chamber time. kappa.max is designed for measurement systems with low timely resolution and limited precision (n = 4, like [29]) but was also tested on a system with higher resolution (n = 11, [30]). Further investigations could be done on the *t*_*meas*_ parameter in the kappa.max decision criteria to improve the application for varying measurement duration. hmr’s estimates of *φ* and *C*_0_ as well as chamber height *h* could be a helpful source of information to test the reliability of the nonlinear fit. There are reasonable arguments that *kappa* should increase with increasing measurement time but not actually decrease as in the kappa.max equation (Eq 2). On the one hand, for shorter measurement times, the chance is higher that the measurement stays in the regime that can be approximated with linear regression, but on the other hand the regression gets more robust with increased time. Starting from our simple suggestion of how to restrict *kappa*, a user may still adjust its calculation procedure to the specific features of his system, using our simulation framework. Especially *t*_*meas*_ can still be tweaked to improve the selection algorithm as this value is not yet perfectly defined. The simulation framework is provided as function to the gasfluxes R package [17].

## Conclusion

Large uncertainties can be introduced by the flux calculation method. The situation of each specific measurement system should be analysed to choose the best combination of linear regression (enhancing bias/reducing uncertainty) and hmr (enhancing uncertainty/reducing bias). We recommend the use of kappa.max approach elaborated in this publication that should optimally balance bias and uncertainty with respect to measurement precision and the chamber setup. We show the practical performance of this FCS decision rule in a representative example dataset. It can be applied without expert tuning or additional field data. The deviation from linear estimates is significantly smaller with kappa.max than other nonlinear methods. Finally, the potential bias and uncertainty of a certain dataset can be estimated with the simulation framework.
